# Psychometric Properties of Language Assessments for Children Aged 4–12 Years: A Systematic Review

**DOI:** 10.3389/fpsyg.2017.01515

**Published:** 2017-09-07

**Authors:** Deborah Denman, Renée Speyer, Natalie Munro, Wendy M. Pearce, Yu-Wei Chen, Reinie Cordier

**Affiliations:** ^1^College of Healthcare Sciences, James Cook University Townsville, QLD, Australia; ^2^Faculty of Health Science, Curtin University Perth, WA, Australia; ^3^Leiden University Medical Centre Leiden, Netherlands; ^4^Faculty of Health Science, The University of Sydney Sydney, NSW, Australia; ^5^School of Allied Health, Australian Catholic University Sydney, NSW, Australia

**Keywords:** language assessment, language impairment, psychometric properties, reliability, validity, Language Disorder

## Abstract

**Introduction:** Standardized assessments are widely used by speech pathologists in clinical and research settings to evaluate the language abilities of school-aged children and inform decisions about diagnosis, eligibility for services and intervention. Given the significance of these decisions, it is important that assessments have sound psychometric properties.

**Objective:** The aim of this systematic review was to examine the psychometric quality of currently available comprehensive language assessments for school-aged children and identify assessments with the best evidence for use.

**Methods:** Using the PRISMA framework as a guideline, a search of five databases and a review of websites and textbooks was undertaken to identify language assessments and published material on the reliability and validity of these assessments. The methodological quality of selected studies was evaluated using the COSMIN taxonomy and checklist.

**Results:** Fifteen assessments were evaluated. For most assessments evidence of hypothesis testing (convergent and discriminant validity) was identified; with a smaller number of assessments having some evidence of reliability and content validity. No assessments presented with evidence of structural validity, internal consistency or error measurement. Overall, all assessments were identified as having limitations with regards to evidence of psychometric quality.

**Conclusions:** Further research is required to provide good evidence of psychometric quality for currently available language assessments. Of the assessments evaluated, the Assessment of Literacy and Language, the Clinical Evaluation of Language Fundamentals-5th Edition, the Clinical Evaluation of Language Fundamentals-Preschool: 2nd Edition and the Preschool Language Scales-5th Edition presented with most evidence and are thus recommended for use.

## Introduction

Language impairment refers to difficulties in the ability to comprehend or produce spoken language relative to age expectations (Paul and Norbury, 2012a). Specific language impairment is defined when the language impairment is not explained by intellectual, developmental or sensory impairments^1^ (American Psychiatric Association, 2013; World Health Organisation, 2015). Specific Language Impairment is estimated to affect 2–10% of school-aged children with variation occurring due to using different diagnostic criteria (Dockrell and Lindsay, 1998; Law et al., 2000; Lindsay et al., 2010). While there is active debate over terminology and definitions surrounding this condition (Ebbels, 2014), according to Bishop (2011), these children present with “unexplained language problems” that require appropriate diagnosis and treatment because of their increased risk of long-term literacy difficulties (Catts et al., 2008; Fraser and Conti-Ramsden, 2008), social-emotional difficulties (Conti-Ramsden and Botting, 2004; McCormack et al., 2011; Yew and O'Kearney, 2013) and poorer academic outcomes (Dockrell and Lindsay, 1998; Conti-Ramsden et al., 2009; Harrison et al., 2009).

Language assessments are used for a range of purposes. These include: initial screening, diagnosis of impairment, identifying focus areas for intervention, decision-making about service delivery, outcome measurement, epidemiological purposes and other research pursuits that investigate underlying cognitive skills or neurobiology (Tomblin et al., 1996; Shipley and McAfee, 2009; Paul and Norbury, 2012b). Whilst few formal guidelines exist, current literature identifies that speech pathologists should use a range of assessment approaches when making judgments about the spoken language abilities of school-aged children, such as: standardized assessment, language-sampling, evaluation of response-to-intervention, dynamic assessment, curriculum-based assessment and caregiver and teacher reports (Reed, 2005; Bishop and McDonald, 2009; Caesar and Kohler, 2009; Friberg, 2010; Hoffman et al., 2011; Haynes and Pindzola, 2012; Paul and Norbury, 2012c; Eadie et al., 2014). Nonetheless, standardized assessments are a widely used component of the assessment process (Hoffman et al., 2011; Spaulding et al., 2012; Betz et al., 2013), particularly for determining if an individual meets diagnostic criteria for Language Impairment (American Psychiatric Association, 2013; World Health Organisation, 2015) and determining eligibility for services (Reed, 2005; Spaulding et al., 2006; Wiig, 2010). Standardized assessments are also designed to be easily reproducible and consistent, and as a result are also widely used in research (Tomblin et al., 1996; Betz et al., 2013).

Language assessments used in clinical practice and research applications must have evidence of sound psychometric properties (Andersson, 2005; Terwee et al., 2012; Betz et al., 2013; Dockrell and Marshall, 2015). Psychometric properties include the overarching concepts of validity, reliability and responsiveness (Mokkink et al., 2010c). This data is typically established by the developers of assessments and are often reported in the administration manuals for individual assessments (Hoffman et al., 2011). When data on psychometric properties is lacking, concerns may arise with the use of assessment results to inform important clinical decisions and the accuracy of reported outcome data in research (Friberg, 2010).

Previous studies have identified limitations with regards to the psychometric properties of spoken language assessments for school-aged children (McCauley and Swisher, 1984; Plante and Vance, 1994; Andersson, 2005; Spaulding et al., 2006; Friberg, 2010). An earlier study published in 1984 (McCauley and Swisher, 1984) examined the manuals of 30 speech and language assessments for children in relation to ten psychometric criteria. These criteria were selected by the authors and included description and size of normative sample, selection of items, normative data provided, concurrent and predictive validity, reliability and description of test administration. The appraisal indicated that only 20% of the 30 examined assessments met half of the criteria with the most assessments meeting only two of the ten criteria. A decade later this information was updated through another study (Plante and Vance, 1994) examining the manuals of pre-school language assessments using the same ten criteria. In this later study, 38% of the 21 examined assessments met half the criteria with most assessments meeting four of the ten criteria.

More recently, literature has focussed on diagnostic accuracy (sensitivity and specificity). Although this information is often lacking in child language assessments, some authors have suggested that diagnostic accuracy should be a primary consideration in the selection of diagnostic language assessments, and have applied the rationale of examining diagnostic accuracy first when evaluating assessments (Friberg, 2010). A study published in 2006 (Spaulding et al., 2006) examined the diagnostic accuracy of 43 language assessments for school-aged children. The authors reported that 33 assessment manuals contained information to calculate mean differences between children with and without language impairment. While nine assessments included sensitivity and specificity data in the manual, only five of these assessments were determined by the authors as having an acceptable level of sensitivity and specificity (80% or higher). In another study published in 2010 (Friberg, 2010), an unspecified number of assessment manuals were examined with nine assessments identified as having an acceptable level of sensitivity and specificity. These nine assessments were then evaluated using 11 criteria based on a modification of the ten criteria used in earlier studies (McCauley and Swisher, 1984; Plante and Vance, 1994). No assessments were found to meet all 11 of the psychometric criteria, however all assessments met 8–10 criteria. The findings from these studies suggest that, while the psychometric quality of assessments appears to have improved over the last 30 years, assessments of children's language may still require further development to improve their psychometric quality.

No previous reviews investigating the psychometric properties of language assessments for children were systematic in identifying assessments for review or included studies published outside of assessment manuals. This is important for two reasons, first, to ensure that all assessments are identified, and second, to ensure that all the available evidence for assessments, including evidence of psychometric properties that was published in peer reviewed journals, is considered when making overall judgments. Previous reviews have also lacked a method of evaluating the methodological quality of the studies selected for review. When evaluating psychometric properties, it is important to consider not only outcomes from studies, but also the methodological quality of studies. If the methodological quality of studies is not sound, then outcomes of studies cannot be viewed as providing psychometric evidence (Terwee et al., 2012). In addition, many of the assessments reviewed in previous studies have since been superseded by newer editions. Older editions are often not printed once new editions are released; therefore, an updated review is needed to examine the evidence for assessments that are currently available to speech-pathologists.

In the time since previous reviews of child language assessments were conducted, research has also advanced considerably in the field of psychometric evaluation (Polit, 2015; Mokkink et al., 2016). In 2010, the Consensus Based Standards for the Selection of Health Status Measurement Instruments (COSMIN) taxonomy (http://www.cosmin.nl) was developed through a Delphi study including fifty-seven international experts from disciplines including psychometrics, epidemiology and clinimetrics (Mokkink et al., 2010b,c). COSMIN aims to improve the selection of health-related measurement instruments by clinicians and researchers through the provision of evidence-based tools for use when appraising studies examining psychometric quality (Mokkink et al., 2016). This includes provision of a checklist (http://www.cosmin.nl/COSMIN%20checklist.html) for rating the methodological quality of studies examining psychometric properties (Terwee et al., 2012). The COSMIN taxonomy and checklist has been utilized in a large number systematic reviews (http://www.cosmin.nl/images/upload/files/Systematic%20reviews%20using%20COSMIN.pdf); however, has not yet been applied in the evaluation of the methodological quality of children's language assessments.

The COSMIN taxonomy describes nine measurement properties relating to domains of reliability, validity and responsiveness. Table 1 provides an overview and definition of all the COSMIN domains and measurement properties (Mokkink et al., 2010c). As the terminology in COSMIN is not always consistent with terms used throughout literature (Terwee et al., 2015), examples of terms that may be used across different studies is also given in this Table.

**Table 1:** COSMIN domains, psychometric properties, aspects of psychometric properties and similar terms based on Mokkink et al. (2010c).

**Domain**	**Psychometric property (definition)**	**Examples of terms used outside of COSMIN that may relate to measurement property**
Reliability	Internal consistency (The degree of the interrelatedness between items)	Internal reliability Content sampling Conventional item analysis
	Reliability (Variance in measurements which is because of “true” differences among clients)	Inter-rater reliability Inter-scorer reliability Test-retest reliability Temporal stability Time sampling Parallel forms reliability
	Measurement error (Systematic and random error of a client's score that is not due to true changes in the construct to be measured)	Standard Error of Measurement
Validity	Content Validity (The degree to which the content of an instrument is an adequate reflection of the construct to be measured)	n/a
	Construct validity (The degree to which scores are consistent with hypotheses based on the assumption that the instrument validly measures the construct to be measured)	n/a
	Aspect of construct validity—structural validity (The degree to which scores reflect the dimensionality of the measured construct)	Internal structure
	Aspect of Construct validity—hypothesis testing (Item construct validity)	Concurrent validity Convergent validity Predictive validity Discriminant validity Contrasted groups validity Identification accuracy Diagnostic accuracy
	Aspect of Construct validity-Cross cultural validity (The degree to which the performance of the items on a translated or culturally adapted instrument are an adequate reflection of the performance of the items of the original version of the instrument)	n/a
	Criterion validity (The degree to which scores reflect measurement from a “gold standard”)	Sensitivity/specificity (when comparing assessment with gold-standard)
Responsiveness	Responsiveness (The ability to detect change over time in the construct to be measured)	Sensitivity/specificity (when comparing two administrations of an assessment) Changes over time Stability of diagnosis
^a^Interpretability	Interpretability (The degree to which qualitative meaning can be assigned to quantitative scores obtained from the assessment)	n/a

a *Interpretability is not considered a psychometric property*.

### Study aim

The aim of this study was to systematically examine and appraise the psychometric quality of diagnostic spoken language assessments for school-aged children using the COSMIN checklist (Mokkink et al., 2010b,c). Specifically, this study aimed to collect information on the overall psychometric quality of assessments and identify assessments with the best evidence of psychometric quality.

## Methods

### Selection criteria

Assessments selected for inclusion in the review were standardized norm-referenced spoken language assessments from any English-speaking country with normative data for use with mono-lingual English-speaking children aged 4–12 years. Only the most recent editions of assessments were included. Initial search results indicated 76 assessments meeting this criterion. As it was not possible to review such a large number of assessments, further exclusion criteria were applied. Assessments were excluded if they were not published within the last 20 years. It is recognized that norm-referenced assessments should only be used with children whose demographics are represented within the normative sample (Friberg, 2010; Paul and Norbury, 2012b; Hegde and Pomaville, 2013); therefore the use of assessments normed on populations from several decades ago may be questionable with current populations. Screening assessments were excluded as they are designed to identify individuals who are at risk or may require further diagnostic assessment (Reed, 2005; Paul and Norbury, 2012b) and thus have a different purpose to diagnostic assessments. Similarly, assessments of academic achievement were also excluded, as although they may assess language ability, this occurs as part of a broad purpose of assessing literacy skills for academic success (Wiig, 2010).

For diagnosis of Specific Language Impairment using standardized testing, previous research has recommended the use of composite scores that include measures of both comprehension and production of spoken language across three domains: word (semantics), sentence (morphology and syntax) and text (discourse) (Tomblin et al., 1996; Gillam et al., 2013). While phonology and pragmatics may also be assessed, these areas are not typically considered part of the diagnostic criteria for identifying Specific Language Impairment (Tomblin et al., 1996). While some evidence suggests that children's language skills may not be contrastive across modalities of comprehension and production (Tomblin and Zhang, 2006; Leonard, 2009), current literature conceptualizes language in this way (Wiig, 2010; World Health Organisation, 2015). A recent survey of SLP's in the United States also identified that “comprehensive” language assessments that assess multiple language areas are used more frequently than assessments that assess a single domain or modality (Betz et al., 2013). As comprehensive assessments provide a broad picture of a child's language strengths and weaknesses, these assessments are often selected first, with further examination of specific domains or modalities conducted if necessary (Betz et al., 2013; Dockrell and Marshall, 2015).

Given the support in literature for the use of comprehensive assessments in diagnostics and the wide use of these assessments by speech pathologists, it was identified that a review of comprehensive language assessments for school-aged children is of particular clinical importance. Therefore, assessments were included in this study if they were the latest edition of a language assessment with normative data for monolingual English speaking children aged 4–12 years; were published within the last 20 years; were primarily designed as a diagnostic assessment; and were designed to assess language skills across at least two of the following three domains of spoken language: word (semantics), sentence (syntax/morphology) and text (discourse).

### Sources of information

**Figure 1:**
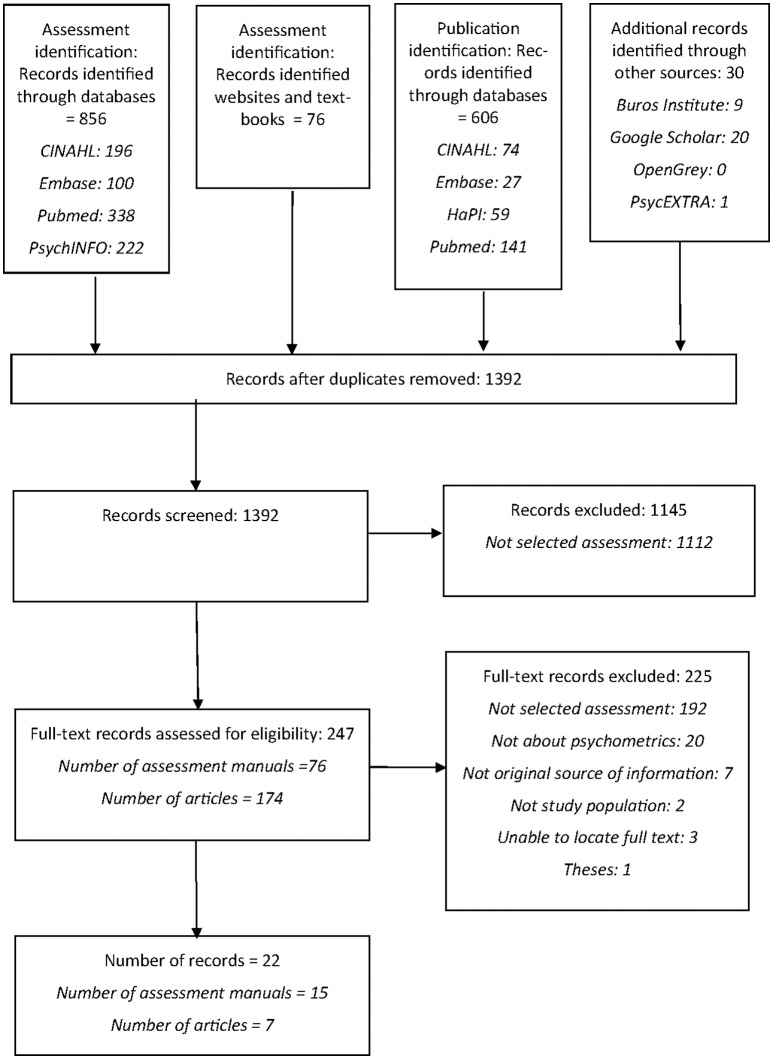
Flowchart of selection process according to PRISMA.

The Preferred Reporting Items for Systematic Reviews and Meta-Analyses (PRISMA) guidelines were developed through consensus of an international group to support high quality reporting of the methodology of systematic reviews (Moher et al., 2009) and were thus used to guide this review. Language assessments were identified through database searches and through comprehensively searching publisher websites, speech pathology websites and textbooks. A flowchart outlining sources of information is contained in Figure 1.

Database searches of PubMed, CINAHL, PsycINFO, and Embase were conducted between February and March 2014. Database searches were conducted with subject headings or mesh terms to identify relevant articles up until the search date. Free text word searches were also conducted for the last year up until the search date to identify recently published articles not categorized in subject headings. The search strategies are described in Table 2.

**Table 2:** Search Terms used in database searches.

	**Database (search date) and search terms**	**Limitations**
**ASSESSMENT IDENTIFICATION**		
Subject Headings	**CINAHL (17.02.14):** [(MH “Psychometrics”) OR (MH “Measurement Issues and Assessments”) OR (MH “Reliability & Validity”)] AND [(MH “Language tests”) OR (MH “Speech and Language Assessment”)]	Child, preschool: 2–5 years; Child: 6–12 years
	**Embase (17.02.14):** (psychometry/OR validity/OR reliability/) AND (Language test/)	English language; Preschool child < 1 to 6 years>; School child < 7 to 12 years>
	**PsycINFO (17.02.14):** [(DE “Psychometrics”) OR (DE “Statistical reliability”) OR (DE “Test reliability”) OR (DE “Statistical validity”) OR (DE “Test validity”)] AND (DE “Language”) AND [(DE “Testing”) OR (DE “Measurement”)]	No limitations
	**PubMed (17.02.14):** (“Psychometrics”[Mesh] OR “Reproducibility of Results”[Mesh]) OR “Validation Studies”[Publication Type] OR “Validation Studies as Topic”[Mesh]) AND (“Language Tests”[Mesh])	(English[lang]) AND (“child”[MeSH Terms:noexp] OR “child, preschool”[MeSH Terms])
Free Text Words	**CINAHL (24.03.14):** (Psychometric^*^ OR Reliability OR Validity) AND (Language OR Speech OR Vocabulary OR Grammar) AND (Measurement^*^ OR Test OR Tests OR Testing OR Assessment^*^ OR Screening^*^)	English language; Child, preschool: 2–5 years; Child: 6–12 years; Publication date: 20130101-20141231
	**Embase (24.03.14):** *As per CINAHL Free Text*	English language; Preschool child < 1 to 6 years>; School child < 7 to 12 years>; yr = “2013-Current”
	**PsycINFO (24.03.14):** *As per CINAHL Free Text*	English; Preschool age (2–5 years); School Age (6–12 years); Adolescence (13–17 years); Publication year: 2013–2014
	**PubMed (17.02.14):** *As per CINAHL Free Text*	English; Preschool Child: 2–5 years; Child: 6–12 years; Publication date from 2013/01/01 to 2014/02/31
Gray Literature	**Google (20:06:15):** (“Speech Pathology” OR “Speech Therapy” OR “Speech Language” AND “Assessment” OR “Test” AND “Publishers” OR “Publishing Companies” OR “textbooks”	No limitations
	**Speechbite (20/06/15):** “Language” AND “Assessment” OR “Test” OR “Psychometrics”	No limitations
**PUBLICATION INDENTIFICATIONS**		
Free Text Words^a^	**CINAHL (20.01.15):** (Name of assessment) OR (Acronym of assessment)	English Language
	**Embase (12.12.14):** *As per CINAHL Free Text*	English language
	**PsycINFO (22.01.15):** *As per CINAHL Free Text*	English
	**PubMed (23.01.15):** *As per CINAHL Free Text*	English
	**HaPI (06.12.14):** *As per CINAHL Free Text*	English
Gray literature^a^	**HaPI (06.12.14):** *As per CINAHL Free Text*	English
	**PsycEXTRA (21/01/15):** (Name of assessment) OR (Acronym of assessment)	Publication year of assessment to current
	**Opengrey (22/01/15):** (Name of assessment) OR (Acronym of assessment)	No limitations
	**Google Scholar (11/01/15):** (Name of assessment) OR (Acronym of assessment)	No limitations

a *The title of the assessment and its acronym were used as search strategy*.

Assessments were also identified from searches of websites and textbooks. Speech pathology association websites from English speaking countries were searched and one website, the American Speech and Hearing Association, was identified as having an online directory of assessments. The website for this directory was identified as being no longer available as of 30/01/16. Publisher websites were identified by conducting Google searches with search terms related to language assessment and publishing and by searching the publisher sites from assessments already identified. These search terms are listed in Table 2. From these methods, a total of 43 publisher websites were identified and searched. Textbooks were identified from Google searches related to language assessment and the contents of recently published books searched. Three recently published textbooks (Kaderavek, 2011; Paul and Norbury, 2012b; Hegde and Pomaville, 2013) were identified as having lists of language assessments, which were then searched for assessments not already identified.

Published articles relating to psychometric properties of selected assessments were identified through additional database searches conducted between December 2014 and January 2015 using PubMed, CINAHL, Embase, PsycINFO, and HaPI. Searches were conducted using full names of assessments as well as acronyms; and limited to articles written in English and published in or after the year the assessment was published. Articles were included in the psychometric evaluation if they related to one of the selected assessments, contained information on reliability and validity and included children speaking English as a first language in the study. Google Scholar, OpenGrey (http://www.opengrey.eu) and PsycEXTRA® (http://www.apa.org/pubs/databases/psycextra/) were also searched for gray literature. Search terms are contained in Table 2.

All retrieved articles were reviewed for inclusion by two reviewers independently using selection criteria, with differences in opinion settled by group discussion to reach consensus. All appropriate articles up until the search dates were included.

### Study selection

Across all searches, a total of 1,395 records were retrieved from databases and other sources. The abstracts for these records were reviewed and 1,145 records were excluded as they were not related to language assessment for mono-lingual English-speaking children aged 4–12 years. The full text versions of remaining records were then reviewed and 225 records were excluded as they did not provide information on the 15 selected assessments, did not contain information on the reliability and validity of selected assessments, did not examine the study population, or were unpublished or unable to be located. Records were also excluded if they were not an original source of information on the reliability and validity of selected assessments. For example, articles reviewing results from an earlier study or reviewing information from an assessment manual were not included if they did not contain new data from earlier studies. A total of 22 records were identified for inclusion, including 15 assessment manuals and 7 articles. Figure 1 represents the assessment and article selection process using a PRISMA flowchart.

### Data collection process and data synthesis

Studies selected for inclusion in the review were rated on methodological quality using COSMIN with the outcome from studies then rated against criteria based on Terwee et al. (2007) and Schellingerhout et al. (2011). Studies for each measurement property for each assessment were then combined to give an overall evidence rating for each assessment using criteria based on Schellingerhout et al. (2011). This methodology is similar to methodology used in previous systematic reviews examining the other health related measurement instruments (Schellingerhout et al., 2011; Uijen et al., 2012; Vrijman et al., 2012).

The four point COSMIN checklist (http://www.cosmin.nl/images/upload/files/COSMIN%20checklist%20with%204-point%20scale%2022%20juni%202011.pdf) was used for rating methodology (Terwee et al., 2012). This checklist provides a system for rating each of the nine COSMIN measurement properties (internal consistency, reliability, measurement error, content validity, structural validity, hypothesis testing, cross-cultural validity, criterion validity and responsiveness). Interpretability can also be measured but is not considered a psychometric property (Mokkink et al., 2009). Each COSMIN measurement property is assessed on 5–18 items that rate the standard of methodological quality using an “excellent,” “good,” “fair,” or “poor” rating scale (Terwee et al., 2012). Items vary depending on the property being rated; however, most properties include ratings for reporting and handling of missing information, sample size, design flaws and type of statistical analysis. There are also property specific items; for example, time interval, patient stability and similarities in testing conditions are rated for test-retest reliability studies.

Different methods for scoring the COSMIN 4-point checklist are employed in studies examining the methodology of psychometric studies. One suggested method is a “worst rating counts” system, where each measurement property is given the score of the item with the lowest rating (Terwee et al., 2012). The advantage of this method over other methods, such as giving a “mean score” for each measurement property, is that serious flaws cannot be compensated for by higher scores on other items (Terwee et al., 2012). However, the “worst rating counts” system is severe as an assessment needs only one “poor” rating to be “poor” for a given measurement property and must receive all “excellent” scores to be rated “excellent” for a measurement property. Previous studies (Speyer et al., 2014) have also identified that this method lacks the ability to distinguish “better” assessments when all reviewed assessments have limitations leading to poor ratings on some items.

In this current study, the scores for each item were “averaged” to give an overall rating for each measurement property. This provides information on the methodological quality in general for studies that were rated. In the scoring process, the appropriate measurement properties were identified and a rated on the relevant items. The options for “excellent,” “good,” “fair,” and “poor” on the 4-point checklist were ranked numerically, with “excellent” being the highest score and “poor” being the lowest score. As the current version of the COSMIN 4 point scale was designed for a “worst rating counts” method, some items do not have options for “fair” or “poor.” Therefore, this was adjusted for in the percentage calculation so that the lowest possible option for each item was considered a 0 score. As each measurement property has a different number of items or may have items that are not applicable to a particular study, the number of items rated may differ across measurement properties or across studies. Therefore, overall scores for each measurement property rated from each study were calculated as a percentage of points received compared to total possible points that a study could have received for that measurement property. The resulting percentages for each measurement property were then classified according to quartile, that is: “Poor” = 0–25%, “Fair” = 25.1–50%, “Good” = 50.1–75%, and “Excellent” = 75.1–100% (Cordier et al., 2015). Where a measurement property was rated “excellent” or “good” overall but had a “poor” score at item level for important aspects such as sample size or statistical analysis, this was noted so that both quantitative scores depicting overall quality and descriptive information about specific methodological concerns may be considered when viewing results.

The findings from studies with “fair” or higher COSMIN ratings were subsequently appraised using criteria based on Terwee et al. (2007) and Schellingerhout et al. (2011). These criteria are described in Table 3. Because the COSMIN ratings were averaged to give a rating of overall quality and Table 3 rates studies against specific methodological criteria, it is possible for studies with good COSMIN ratings in to be rated as indeterminate from Table 3.

**Table 3:** Criteria for measuring quality of findings for studies examining measurement properties based on Terwee et al. (2007) and Schellingerhout et al. (2011).

**COSMIN measurement property**	**Rating**	**Quality Criteria**
Internal consistency	+	Subtests one-dimensional (determined through factor analysis with adequate sample size) and Cronbach alpha between 0.70 and 0.95
	?	Dimensionality of subtests unknown (no factor analysis) or Cronbach's alpha not calculated
	−	Subtests uni-dimensional (determined through factor analysis with adequate sample size) and Cronbach's alpha < 0.7 or > 0.95
	±	Conflicting results
	NR	No information found on internal consistency
	NE	Not evaluated due to “poor” methodology rating on COSMIN
Reliability	+	ICC/weighted Kappa equal to or > than 0.70
	?	Neither ICC/weighted Kappa calculated or doubtful design or method (e.g., time interval not appropriate)
	−	ICC/weighted Kappa < 0.70 with adequate methodology
	±	Conflicting results
	NR	No information found on reliability
	NE	Not evaluated due to “poor” methodology on COSMIN
Measurement error	+	MIC > SDC or MIC equals or inside LOA
	?	MIC not defined or doubtful design or method
	−	MIC < SDC or MIC equals or inside LOA with adequate methodology
	+	Conflicting results
	NR	No information found on measurement error
	NE	Not evaluated due to “poor” methodology on COSMIN
Content validity	+	Good methodology (i.e., an overall rating of “Good” or above on COSMIN criteria for content validity) and experts examined all items for content and cultural bias during development of assessment
	?	Questionable methodology or experts only employed to examine one aspect (e.g., cultural bias)
	−	No expert reviewer involvement
	±	Conflicting results
	NR	No information found on content validity
	NE	Not evaluated due to “poor” methodology
Structural validity	+	Factor analysis performed with adequate sample size. Factors explain at least 50% of variance
	?	No factor analysis or inadequate sample size. Explained variance not mentioned
	−	Factors explain < 50% of variance despite adequate methodology
	±	Conflicting results
	NR	No information found on structural validity
	NE	Not evaluated due to “poor” methodology
Hypothesis testing	+	Convergent validity: Correlation with assessments measuring similar constructs equal to or >0.5 and correlation is consistent with hypothesis Discriminant validity: findings consistent with hypotheses using appropriate statistical analysis (e.g., *t*-test *p* < 0.05 or Cohen's d effect size > 0.5)
	?	Questionable methodology e.g., only correlated with assessments that are not deemed similar
	−	Discriminant validity: findings inconsistent with hypotheses (e.g., no significant difference identified from appropriate statistical analysis) Convergent validity: Correlation with assessments measuring similar constructs equal to or < 0.5 or correlation is inconsistent with hypothesis
	±	Conflicting results
	NR	No information found on hypothesis testing
	NE	Not evaluated due to “poor” methodology

*+, Positive result; −, Negative result; ?, Indeterminate result due to methodological shortcomings; ±, Conflicting results within the same study (e.g., high correlations for some results but not on others); NR, Not reported; NE, Not evaluated; MIC, minimal important change; SDC, smallest detectable change; LOA, limits of agreement; ICC, Intra-class correlation; SD, standard deviation*.

Overall evidence ratings for each measurement property for each assessment were then determined by considering available evidence from all the studies. These ratings were assigned based on quality of methodology available studies (as rated using COSMIN) and the quality of the findings from the studies (as defined in Table 3). This rating scale was based on criteria used by Schellingerhout et al. (2011) and is outlined in Table 4.

**Table 4:** Level of evidence for psychometric quality for each measurement property based on Schellingerhout et al. (2011).

**Level of evidence**	**Rating**	**Criteria based on appraisal of quality of methodology (rated according to COSMIN) and quality of findings (rated according to Table 3)**
Strong evidence	+++ or −−−	Consistent findings across 2 or more studies of “good” methodological quality OR one study of “excellent” methodological quality
Moderate evidence	++ or −−	Consistent findings across 2 or more studies of “fair” methodological quality OR one study of “good” methodological quality
Weak evidence	+ or −	One study of “fair” methodological quality (examining convergent or discriminant validity if rating hypothesis testing)
Conflicting evidence	±	Conflicting findings across different studies (i.e., different studies with positive and negative findings)
Unknown	?	Only available studies are of “poor” methodological quality
Not Evaluated	NE	Only available studies are of “poor” methodological quality as rated on COSMIN

*+, Positive result; –, Negative result*.

To limit the size of this review, selected assessments were not appraised on the measurement property of responsiveness, as that would have significantly increased the size of the review. Interpretability is not considered a psychometric property and was also not reviewed. However, given the clinical importance of responsiveness and interpretability, it is recommended that these properties be a target for future research. Cross-cultural validity applies when an assessment has been translated or adapted from another language. As all the assessments reviewed in this study were originally published in English, cross-cultural validity was not rated. However, it is acknowledged that the use of English language assessments with the different dialects and cultural groups that exist across the broad range of English speaking countries is an area that requires future investigation. Criterion validity was also not evaluated in this study as this measurement property refers to a comparison of an assessment to a diagnostic “gold-standard” (Mokkink et al., 2010a). Consultation with experts and reference to current literature (Tomblin et al., 1996; Dollaghan and Horner, 2011; Betz et al., 2013) did not identify a “gold-standard” or an industry recognized “reference standard” for diagnosis of language impairment, therefore all studies comparing one assessment to another assessment were considered convergent validity and rated as hypothesis testing according to COSMIN.

Diagnostic accuracy, which includes sensitivity and specificity and positive predictive power calculations, is an area that does not clearly fall into a COSMIN measurement property. However, current literature identifies this as being an important consideration for child language assessment (Spaulding et al., 2006; Friberg, 2010). In this review, data from studies examining diagnostic accuracy was collated in a **Table 9** to allow for this information be considered alongside information on COSMIN measurement properties. It should be noted that these studies were not rated for methodological quality, as the COSMIN checklist was not identified as providing an appropriate rating scale for these types of studies. However, descriptive information on the methodological quality of these studies was commented upon in the results section.

Where several studies examining one measurement property were included in a manual, one rating was provided based on information from the study with the best methodology. For example, if a manual included internal consistency studies using different populations then a rating for internal consistency was given based on the study with the most comprehensive or largest sample size. The exception was for reliability, where test-retest and inter-rater reliability were rated separately and hypothesis testing where convergent validity and discriminant validity were rated separately. In most cases, these different reliability and hypothesis testing studies were conducted using different sample sizes and different statistical analyses. As it was considered that manuals that include both these studies for each measurement property are providing evidence across different aspects of the measurement property, it was decided that counting these as different studies would allow this to be reflected in final data.

Some assessments also included studies for hypothesis testing examining gender, age and socio-cultural differences. Whilst this information contributes important information on an assessment's usefulness, we identified convergent validity and discriminant validity as key aspects for the measurement property of hypothesis testing and thus only included these studies in this review.

### Risk of bias

All possible items for each assessment were rated from all identified publications. Where an examination of a particular measurement property was not reported in a publication or not reported with enough detail to be rated, this was rated as “not reported” (NR). Two raters were involved in appraising publications. To ensure consistency, both raters involved in the study trained as part of a group prior to rating the publications for this study. The first rater rated all publications with a random sample of 40% of publications also rated independently by a second rater. Inter-rater reliability between the two raters was calculated and determined to be adequate (weighted Kappa = 0.891; SEM = 0.020; 95% confidence interval = 0.851–0.931). Any differences in opinion were discussed and the first rater then appraised the remaining 60% of articles applying rating judgments agreed upon after consensus discussions.

## Results

### Assessments selected for review

A total of 22 publications were identified for inclusion in this review. These included 15 assessment manuals and seven journal articles relating to a total of 15 different assessments. From the 22 publications, 129 eligible studies were identified, including three studies that provided information on more than one of the 15 selected assessments. Eight of these 129 studies reported on diagnostic accuracy and were included in the review, but were not rated using COSMIN, leaving 121 articles to be rated for methodological quality. Of the 15 selected assessments, six were designed for children younger than 8 years and included the following assessments: Assessment of Literacy and Language (ALL; nine studies), Clinical Evaluation of Language Fundamentals: Preschool-2nd Edition (CELF:P-2; 14 studies), Reynell Developmental Language Scales-4th Edition (NRDLS; six studies), Preschool Language Scales-5th Edition (PLS-5; nine studies), Test of Early Language Development-3rd Edition (TELD-3; nine studies) and Test of Language Development-Primary: 4th Edition (TOLD-P:4; nine studies). The Test of Language Development-Intermediate: 4th Edition (TOLD-I:4; nine studies) is designed for children older than 8 years. The remaining eight assessments covered most of the 4–12 primary school age range selected for this study and included the following assessments: Assessment of Comprehension and Expression (ACE 6-11; seven studies), Comprehensive Assessment of Spoken Language (CASL; 12 studies), Clinical Evaluation of Language Fundamentals-5th Edition (CELF-5; nine studies), Diagnostic Evaluation of Language Variance-Norm Referenced (DELV-NR; ten studies), Illinois Test of Psycholinguistic Abilities-3rd Edition (ITPA-3; eight studies), Listening Comprehension Test-2nd Edition (LCT-2; seven studies), Oral and Written Language Scales-2nd Edition (OWLS-2; eight studies) and Woodcock Johnson 4th Edition Oral Language (WJIVOL; six studies). These 15 selected assessments are summarized in Table 5 with regards to author, publication date and language area assessed.

**Table 5:** Summary of assessments included in the review.

**Acronym and Name of Test (Authors; Publication date)**	**Age-group**	**Areas assessed Subtests (norm-referenced) Composite scores derived from subtests**
**ACE6-11** Assessment of Comprehension and Expression 6–11 (Adams et al., 2001)	6–11 years	Spoken language including pragmatics. Subtests: • Sentence comprehension • Inferential comprehension • Naming • Syntactic formulation • Semantic decisions • Non-Literal comprehension • Narrative propositions • Narrative syntax/discourse Composite Scores: • Overall Language Score (Main Test or Extended version)
**ALL** Assessment of Literacy and Language (Lombardino et al., 2005)	^a^Pre-school—grade 1	Spoken and written language skills including phonemic awareness Subtests: • Letter Knowledge • Rhyme Knowledge • Basic Concepts • Receptive Vocabulary • Parallel Sentence Production • Ellison • Word Relationships • Rhyme Knowledge • Phonics Knowledge • Sound Categorization • Sight Word Recognition • Listening Comprehension Composite Scores: • Emergent Literacy Index • Language Index • Phonological Index • Phonological-Orthographic Index
**CASL** Comprehensive Assessment of Spoken Language (Carrow-Woolfolk, 1999)	3–21 years	Spoken language including pragmatics Subtests: • Comprehension of Basic Concepts • Antonyms • Synonyms • Sentence Completion • Idiomatic Language • Syntax Construction • Paragraph Comprehension of Syntax • Grammatical Morphemes • Sentence Comprehension of Syntax • Grammaticality Judgment • Non-Literal Language • Meaning from Context • Inference • Ambiguous Sentences • Pragmatic Judgment Composite Scores: • Core Language • Lexical/Semantic (7;0–21 years only) • Syntactic (7;0–21 years only) • Supra-linguistic (7;0–21 years only) • Receptive Index (7;0–10;11 years only) • Expressive Index (7;0–10;11 years only)
**CELF-5** Clinical Evaluations of Language Fundamentals—5th Edition (Wiig et al., 2013)	5;0–21;11 years	Spoken language; supplemental tests for reading, writing and pragmatics Subtests: • Sentence Comprehension • Linguistic Concepts • Word Structure • Word Classes • Following Directions • Formulated Sentences • Recalling Sentences • Understanding Spoken Paragraphs • Word Definitions • Sentence Assembly • Semantic Relationships • Sentence Comprehension • Reading Comprehension (not used in composite scores) • Structured Writing (not used in composite scores) • Pragmatics profile (observational checklist, not used in composite scores) Composite Scores: • Core Language • Receptive Language • Expressive Language • Language Content • Language Structure • Language Memory
**CELF-P:2** Clinical Evaluation of Language Fundamentals: Preschool—2nd Edition (Wiig et al., 2004)	3;0–6;11 years	Spoken language Subtests: • Sentence Structure • Word Structure • Expressive Vocabulary • Concepts and Following Directions • Recalling Sentences • Basic Concepts • Word Classes Composite Scores: • Core Language • Receptive Language • Expressive Language • Language Content • Language Structure • Recalling Sentences in Context (not used in composite scores) • Phonological Awareness (not used in composite scores) • Pre-Literacy Rating Scale (not used in composite scores)
**DELV-NR** Diagnostic Evaluation of Language Variation—Norm referenced (Seymour et al., 2005)	4–9 years	Spoken language: Subtests: • Semantics • Syntax • Pragmatics • Phonology (not used in composite score) Composite Scores: • Total Language Score
**ITPA-3** Illinois Test of Psycholinguistic Abilities—3rd Edition (Hammill et al., 2001)	5;0–12;11 years	Spoken and written language: Subtests: • Spoken Analogies • Spoken Vocabulary • Morphological Closure • Syntactic Sentences • Sound Deletion • Rhyming Sequences • Sentence Sequencing • Written Vocabulary • Sight Decoding • Sound Decoding • Sight Spelling • Sound Spelling Composite Scores: • General Language • Spoken Language • Written Language • Semantics • Grammar • Phonology • Comprehension • Word Identification • Spelling • Sight-Symbol Processing • Sound-Symbol Processing
**LCT-2** The Listening Comprehension Test—2nd Edition (Bowers et al., 2006)	6–11 years	Spoken language Subtests: • Main Idea • Details • Reasoning • Vocabulary • Understanding Messages Composite Score: • Total Score
**NRDLS** Reynell Developmental Language Scale—4th Edition (Edward et al., 2011)	3;0–7;5 years	Spoken language Subtests: • Comprehension • Production Composite Scores: • Total Language Score
**OWLS-II** Oral and Written Language Scales—2nd Edition (Carrow-Woolfolk, 2011)	3–21 years	Spoken language Subtests: • Listening Comprehension • Oral Expression • Reading Comprehension • Written Language Composite Scores: • Oral Language • Written Language • Receptive Language • Expressive Language • Overall Language
**PLS-5** Preschool Language Scales-5th Edition (Zimmerman et al., 2011)	Birth-7;11 years	Spoken language Subtests: • Auditory Comprehension • Expressive Communication Composite Scores: • Total Language Score
**TELD-3** Test of Early Language Development—3rd Edition (Hresko et al., 1999)	3;0–7;11	Spoken language Subtests: • Receptive Language • Expressive Language Composite Scores: • Spoken Language
**TOLD-I:4** Test of Language Development—Intermediate: 4th Edition (Newcomer and Hammill, 2008)	8;0–17 years	Spoken language Subtests: • Sentence Combining • Picture Vocabulary • Word Ordering • Relational Vocabulary • Morphological Comprehension • Multiple Meanings • Word Discrimination (not used in composite scores) • Phonemic Analysis (not used in composite scores) • Word Articulation (not used in composite scores) Composite Scores: • Listening • Organizing • Speaking • Grammar • Semantics • Spoken Language
**TOLD-P:4** Test of Language Development—Primary: 4th Edition (Hammill and Newcomer, 2008)	4;0–8;11 years	Spoken language Subtests: • Sentence Combining • Picture Vocabulary • Word Ordering • Relational Vocabulary • Morphological Comprehension • Multiple Meanings Composite Scores: • Listening • Organizing • Speaking • Grammar • Semantics • Spoken Language
**WJIVOL** Woodcock Johnson IV Tests of Oral Language (Shrank et al., 2014)	2–90 years	Spoken language Subtests: • Picture Vocabulary • Oral Comprehension • Segmentation • Rapid Picture Naming • Sentence Repetition • Understanding Directions • Sound Blending • Retrieval Fluency • Sound Awareness Composite Scores: • Oral Language • Broad Oral Language • Oral Expression • Listening Comprehension • Phonetic coding • Speed of Lexical Access

a *Normative data is based on U.S. school grade level. No normative data is provided for age level in this assessment*.

During the selection process, 61 assessments were excluded as not meeting the study criteria. These assessments are summarized in Table 6 with regards to author, publication date, language area assessed and reason for exclusion.

**Table 6:** Summary of assessments excluded from the review.

	**Name of Test**	**Author and publication date**	**Age-group (years)**	**Language area/s tested**	**Reasons for exclusion**
1	Adolescent Language Screening Test (ALST)	Morgan and Gillford (1984)	11–17	Pragmatics, receptive vocabulary, expressive vocabulary, sentence formulation, morphology and phonology	Not published within last 20 years
2	Aston Index Revised (Aston)	Newton and Thomson (1982)	5–14	Receptive language, written language, reading, visual perception, auditory discrimination	Not published within last 20 years
3	Bracken Basic Concept Test-Expressive (BBCS:E)	Bracken (2006)	3–6;11	Expressive: basic concepts	Not comprehensive language assessment
4	Bracken Basic Concept Test-3rd Edition Receptive (BBCS:3-R)	Bracken (2006)	3–6;11	Receptive: basic concepts	Not comprehensive language assessment
5	Bankson Language Test-Second Edition (BLT-2)	Bankson (1990)	3;0–6;11	Semantics, syntax/morphology and pragmatics	Not published within last 20 years
6	Boehm Test of Basic concepts-3rd Edition (Boehm-3)	Boehm (2000)	Grades K-2 (US)	Basic concepts	Not comprehensive language assessment
7	Boehm Test of Basic Concepts Preschool-3rd Edition (Boehm-3 Preschool)	Boehm (2001)	3;0–5;11	Relational concepts	Not comprehensive language assessment
8	British Vocabulary Scale-3rd Edition (BPVS-3)	Dunn et al. (2009)	3–16	Receptive vocabulary	Not comprehensive language assessment
9	Clinical Evaluation of Language Fundamentals–5th Edition Metalinguistics (CELF-5 Metalinguistic)	Wiig and Secord (2013)	9;0–21;0	Higher level language: making inferences, conversation skills, multiple meanings and figurative language	Not comprehensive language assessment
10	Clinical Evaluations of Language Fundamentals-5th Edition Screening (CELF-5 Screening)	Semel et al. (2013)	5;0–21;11	Receptive and expressive semantics and syntax	Screening assessment
11	Comprehensive Receptive and Expressive Vocabulary Test-Second Edition (CREVT-3)	Wallace and Hammill (2013)	5–89	Receptive and expressive vocabulary	Not comprehensive language assessment
12	Compton Speech and Language Screening Evaluation-Revised Edition	Compton (1999)	3–6	Expressive and receptive language, articulation, auditory memory and oral-motor co-ordination	Screening Assessment
13	Executive Functions Test Elementary	Bowers and Huisingh (2014)	7;0–12;11	Higher level language: working memory, problem solving, inferring and making predictions	Not comprehensive language assessment
14	Expressive Language Test-2nd Edition (ELT-2)	Bowers Huisingh et al. (2010)	5;0–11;0	Expressive language: sequencing, metalinguistics, grammar and syntax	Not comprehensive language assessment
15	Expressive One-Word Vocabulary Test-4th Edition (EOWPVT-4)	Martin and Brownell (2011)	2–80	Expressive vocabulary (picture naming)	Not comprehensive language assessment
16	Expression, Reception and Recall of Narrative Instrument (ERRNI)	Bishop (2004)	4–15	Narrative skills: story comprehension and retell	Not comprehensive language assessment
17	Expressive Vocabulary Test-Second Edition (EVT-2)	Williams (2007)	2;6–90+	Expressive vocabulary and word retrieval	Not comprehensive language assessment
18	Fluharty Preschool Screening Test-Second Edition (FPSLST-2)	Fluharty (2000)	3;0–6;11	Receptive and expressive language: sentence repetition, answering questions, describing actions, sequencing events and articulation.	Screening Assessment
19	Fullerton Language Test for Adolescent-Second Edition (FLTA-2)	Thorum (1986)	11-Adult	Receptive and expressive language	Not published within last 20 years
20	Grammar and Phonology Screening Test (GAPS)	Van der Lely (2007)	3;5–6;5	Grammar and pre reading skills	Not Comprehensive language assessment
21	Kaufman Survey of Early Academic and Language Skills (K-SEALS)	Kaufman and Kaufman (1993)	3;0–6;11	Expressive and receptive vocabulary, numerical skills and articulation	Not published in last 20 years
22	Kindergarten Language Screening Test-Second Edition (KLST-2)	Gauthier and Madison (1998)	3;6–6;11	General language: question comprehension, following commands, sentence repetition, comparing and contrasting objects and spontaneous speech	Screening Assessment
23	Language Processing Test 3 Elementary (LPT-3:P)	Richard and Hanner (2005)	5–11	Expressive semantics: word association, categorizing words, identifying similarities between words, defining words, describing words	Not comprehensive language assessment
24	Montgomery Assessment of Vocabulary Acquisition (MAVA)	Montgomery (2008)	3–12	Receptive and expressive vocabulary	Not comprehensive language assessment
25	North Western Syntax Screening Test (NSST)	Lee (1969)	Unknown	Syntax and morphology	Not published in last 20 years
26	Peabody Picture Vocabulary test-4th Edition (PPVT-IV)	Dunn and Dunn (2007)	2;6–90	Receptive vocabulary	Not comprehensive language assessment
27	Pragmatic Language Skills (PLSI)	Gillam and Miller (2006)	5;0–12;11	Pragmatics	Not comprehensive language assessment
28	Preschool Language Assessment Instrument-Second Edition (PLAI-2)	Blank et al. (2003)	3.0–5;11	Discourse	Not comprehensive language assessment
29	Preschool Language Scales-5th Edition Screener (PLS-5 Screener)	Zimmerman (2013)	Birth-7;11	General language	Screening assessment
30	Receptive One-Word Picture Vocabulary Tests-Fourth Edition (ROWPVT-4)	Martin and Brownell (2010)	2;0–70	Receptive vocabulary	Not comprehensive language assessment
31	Renfrew Action Picture Test-Revised (RAPT-Revised)	Renfrew (2010)	3–8	Expressive language: information content, syntax and morphology	Not comprehensive language assessment
32	Renfrew Bus Story-Revised edition (RBS-Revised)	Renfrew (2010)	3–8	Narrative retell	Not comprehensive language assessment
33	Rhode Island Test of Language Structure	Engen and Engen (1983)	3–6	Receptive syntax (designed for hearing impairment but has norms for non-hearing impairment)	Not comprehensive language assessment
34	Screening Kit of Language Development (SKOLD)	Bliss and Allen (1983)	2–5	General language	Not published within last 20 years
35	Screening Test for Adolescent Language (STAL)	Prather and Breecher (1980)	11–18	General language	Not published in last 20 years
36	Social Emotional Evaluation (SEE)	Wiig (2008)	6;0–12;0	Social skills and higher level language	Not comprehensive language assessment
37	Social Language Development Test Elementary (SLDT-E)	Bowers et al. (2008)	6–11	Language for social interaction	Not comprehensive language assessment
38	Structured Photographic Expressive Language Test-Third Edition (SPELT-3)	Dawson and Stout (2003)	4,0–9,11	Expressive syntax and morphology	Not comprehensive language assessment
39	Structured Photographic Expressive Language Test Preschool-2nd Edition (SPELT-P:2)	Dawson et al. (2005)	3;0–5;11	Expressive syntax and morphology	Not comprehensive language assessment
40	Test for Auditory Comprehension of Language-Fourth Edition (TACL-4)	Carrow-Woolfolk (2014)	3;0–12;11	Receptive vocabulary, syntax and morphology	Not comprehensive language assessment
41	Test of Auditory Reasoning and processing skills (TARPS)	Gardner (1993)	5–13;11	Auditory processing: verbal reasoning, inferences, problems solving, acquiring and organizing information	Not published within last 20 years
42	Test for Examining Expressive Morphology (TEEM)	Shipley (1983)	3;0–7;0	Expressive morphology	Not published within last 20 years
43	Test of Grammatical Impairment (TEGI)	Rice and Wexler (2001)	3;0–8;0	Syntax and morphology	Not comprehensive language assessment
44	Test of Grammatical Impairment-Screener (TEGI-Screener)	Rice and Wexler (2001)	3–6;11	Syntax and morphology	Screening assessment
45	Test of Language Competence-Expanded (TLC-E)	Wiig and Secord (1989)	5;0–18;0	Semantics, syntax and pragmatics	Not published within last 20 years
46	Test of Narrative language (TNL)	Gillam and Pearson (2004)	5;0–11;11	Narrative retell	Not comprehensive language assessment
47	Test of Pragmatic Language (TOLP-2)	Terasaki and Gunn (2007)	6;0–18;11	Pragmatic skills	Not comprehensive language assessment
48	Test of Problem Solving 3 Elementary (TOPS-3-Elementary)	Bowers et al. (2005)		Language-based thinking	Not comprehensive language assessment
49	Test of Reception of Grammar (TROG-2)	Bishop (2003)	4+	Receptive grammar	Not comprehensive language assessment
50	Test of Semantic Skills-Intermediate (TOSS-I)	Huisingh et al. (2004)	9–13	Receptive and expressive semantics	Not comprehensive language assessment
51	Test of Semantic Skills-Primary (TOSS-P)	Bowers et al. (2002)	4–8	Receptive and expressive semantics	Not comprehensive language assessment
52	Test of Word Finding-Second Edition (TWF-2)	German (2000)	4;0–12;11	Expressive vocabulary: word finding	Not comprehensive assessment
53	Test of Word Finding in Discourse (TWFD)	German (1991)	6;6–12;11	Word finding in discourse	Not comprehensive assessment
54	Test of Word Knowledge (TOWK)	Wiig and Second (1992)	5–17	Receptive and expressive vocabulary	Not published within last 20 years
55	Token Test for Children-Second edition (TTFC-2)	McGHee et al. (2007)	3;0–12;11	Receptive: understanding of spoken directions	Not comprehensive language assessment
56	Wellcomm: A speech and language toolkit for the early years (Screening tool) English norms	Sandwell Primary Care Trust	6 months–6 years	General language	Screening Assessment
57	Wh—question comprehension test	Vicker (2002)	4-Adult	Wh-question comprehension	Not comprehensive language assessment
58	Wiig Assessment of Basic Concepts (WABC)	Wiig (2004)	2;6–7;11	Receptive and expressive: basic concepts	Not comprehensive assessment
59	Word Finding Vocabulary Test-Revised Edition (WFVT)	Renfrew (2010)	3–8	Expressive vocabulary: word finding	Not comprehensive language assessment
60	The WORD Test 2 Elementary (WORD-2)	Bowers et al. (2004)	6–11	Receptive and expressive vocabulary	Not comprehensive language assessment
61	Utah Test of Language Development (UTLD-4)	Mecham (2003)	3;0–9;11	Expressive semantics, syntax and morphology	Not comprehensive language assessment

The seven identified articles were sourced from database searches and gray literature. These included studies investigating structural and convergent validity (hypothesis testing) of the CASL (Reichow et al., 2008; Hoffman et al., 2011), convergent validity (hypothesis testing) using the CELF-P:2 and the DELV-NR (Pesco and O'Neill, 2012), convergent validity (hypothesis testing) of the CELF-P:2 (Kaminski et al., 2014), convergent validity (hypothesis testing) of the TELD-3 (Spaulding, 2012), diagnostic accuracy of the CELF-P (Eadie et al., 2014), and internal consistency and test-retest reliability of the CASL pragmatic judgment subtest (McKown et al., 2013). All articles appeared to be have been published by authors independent of the developers of the assessments. The seven included articles are described in Table 7.

**Table 7:** Articles selected for review.

**Author**	**Assessment**	**COSMIN property rated from study**
Eadie et al., 2014	CELF-P:2 (Australian) Diagnostic accuracy	Investigation of sensitivity and specificity of CELF:P-2 at age 4 years against Clinical Evaluation of Language Fundamentals-4th Edition (CELF-4) at age 5 years
Hoffman et al., 2011	CASL Structural Validity Hypothesis testing	Investigation of the construct (structural) validity of the CASL using factor analysis. Investigation of convergent validity between the CASL and Test of Language Development-Primary: 3rd Edition (TOLD-P:3)
Kaminski et al., 2014	CELF-P:2 Hypothesis testing	Investigation of predictive validity and convergent validity between CELF:P-2 and Preschool Early Literacy Indicators (PELI)
McKown et al., 2013^*^	CASL Internal consistency Reliability (test-retest)	Examination of the internal consistency of the Pragmatic Judgment subtest of the CASL Examination of test-retest reliability of the Pragmatic Judgment subtest of the CASL
Pesco and O'Neill, 2012	CELF:P-2 DELV-NR Hypothesis testing	Investigation of performance on the DELV-NR and CELF:P-2 to be predicted by the Language Use Inventory (LUI)
Reichow et al., 2008	CASL Hypothesis testing	Examination of the convergent validity between selected subtests from the CASL with the Vineland Adaptive Behavior Scales
Spaulding, 2012	TELD-3 Hypothesis testing	Investigation of consistency between severity classification on the TELD-3 and the Utah Test of Language Development-4th Edition (UTLD-4)

* *This subtest forms part of the overall composite score on the CASL*.

The assessment manuals for all the selected assessments were not available through open sources and were only accessible by purchasing the assessment. Only three published articles by authors of assessments were identified. One of these contained information on the development, standardization and psychometric properties of the NRDLS (Letts et al., 2014). This study was not included in this review as it was published after the assessment manual and contained no new information. Similarly, another article by the developers of the NRLDS (Letts et al., 2013) examined the relationship between the NRDLS scores and economic status. This study was also reported in the manual and was not included. One other study by Seymour and Zurer-Pearson (2004) described the rationale and proposed structure for the DELV-NR assessment; however, this study was also not included as it did not contain information on the psychometric properties of the final version of the assessment.

### Psychometric evaluation

The results of the COSMIN ratings of the psychometric quality of the 15 assessments are listed in Table 8. Thirteen of the 15 assessment manuals included studies on the six COSMIN measurement properties evaluated in this review. One assessment (NRDLS) presented no examination of structural validity and another assessment (WJIVOL) did not have a reliability study using the subtests that primarily contribute to overall composite language scores. Manuals that contained studies with more than one reliability study i.e., inter-rater or test-retest reliability were given a rating for each type of reliability. Similarly, manuals with more than one study of hypothesis testing i.e., convergent or discriminant validity were given more than one ratings for hypothesis testing. This is noted in Table 7 with two ratings for reliability and hypothesis testing where multiple studies were identified.

**Table 8:** Ratings of methodological quality and study outcome of reliability and validity studies for selected assessments.

**Assessment**	**Manual or article**	**Internal consistency**	**Reliability**	**Error measurement**	**Content validity**	**Structural validity**	**Hypothesis testing**
ACE6-11	ACE6-11 Manual	77.8^a^ Excell ?	Test-retest 75.9 Excell ?	53.3^d^ Good ?	42.9 Fair ?	25^h^ Poor NE	Convergent 52.2 Good + Discriminant 23.5 Poor NE
ALL	ALL Manual	75.0^b^ Good ?	Test-retest 72.4 Good ? Inter-rater 50^c^ Fair ?	20^d^ Poor NE	92.9 Excell +	33.3^b^ Fair ?	Convergent 52.2 Good + Discriminant 52.9 Good +
CASL	CASL Manual	57.1^g^ Good ?	Test-retst 56.0^e^ Good ?	40^d^ Fair ?	71.4 Good ?	33.3^b^ Fair ?	Convergent 39.1 Fair + Discriminant 58.8 Good +
	Hoffman et al., 2011	NR	NR	NR	NR	33.3^b^ Fair ?	Convergent 73.9 Good ±
	McKown et al., 2013	83.3^a^ Excell ?	Test-retest 62.0^e^ Good ?	NR	NR	NR	NR
	Reichow et al., 2008	NR	NR	NR	NR	NR	Convergent 52.2 Good ?
CELF-5	CELF-5 Manual	71.4^g^ Good ?	Test-retest 72.4 Good ? Inter-rater 66.7 Good +	40^d^ Fair ?	71.4 Good +	58.3 Good ?	Convergent 65.2 Good + Discriminant 52.9 Good +
CELF:P-2	CELF:P-2 Manual	71.4^b^ Good ?	Test-retest 72.4 Good ? Inter-rater 50.0^c^ Fair ?	40^d^ Fair ?	64.3 Good +	33.3^b^ Fair ?	Convergent 47.8 Fair + Discriminant 58.8 Good +
	Kaminski et al., 2014	NR	NR	NR	NR	NR	Convergent 56.5 Good ±
	Pesco and O'Neill, 2012	NR	NR	NR	NR	NR	Convergent 47.8 Good ±
	^*^Manual for ALL	NR	NR	NR	NR	NR	Convergent 65.2 Good +
	^*^Manual for PLS-5	NR	NR	NR	NR	NR	Convergent 69.6 Good +
DELV-NR	DELV-NR Manual	66.7^a^ Good ?	Test-retest 69 Good ? Inter-rater 50^c^ Fair ?	40^d^ Fair ?	57.1 Good ?	50^h^ Fair ?	Convergent 34.8 Fair ? Discriminant 41.2 Fair ?
	^*^Pesco and O'Neill, 2012	NR	NR	NR	NR	NR	Convergent 47.8 Good ±
ITPA-3	ITPA-3 Manual	71.4^b^ Good ?	Test-retest 62.1 Good ? Inter-rater 41.7 Fair ?	40^d^ Fair ?	57.1 Fair ?	50 Fair ?	Convergent 34.7 Fair + Discriminant 41.2 Fair ?
LCT-2	LCT-2 Manual	50^a^ Fair ?	Test-retest 34.6 Fair ? Inter-rater 25^c^ Poor NE	40^d^ Fair ?	28.5 Fair ?	50^h^ Fair ?	Discriminant 29.4^f^ Fair +
NRDLS	NRDLS Manual	66.7^a^ Good ?	Test-retest 60.0 Good ?	40.0^d^ Fair ?	57.1 Good ?	NR	Convergent 52.2 Good + Discriminant 35.3 Fair +
OWLS-II	OWLS-II Manual	57.1^g^ Good ?	Test-retest 72.4 Good ? Inter-rater 50 Fair +	40^d^ Fair ?	71.4 Good ?	33.4^b^ Fair ?	Convergent 21.7 Poor NR Discriminant 47.1 Fair +
PLS-5	PLS-5 Manual	50^g^ Fair ?	Test-retest 69.0 Good ? Inter-rater 50^g^ Fair ?	40^d^ Fair ?	71.4 Good ?	57.1^h^ Good ?	Convergent 56.5 Good + Discriminant 52.9 Good +
TELD-3	TELD-3 Manual	61.1^a^ Good ?	Test-retest 72.4 Good ? Inter-rater 33.3^g^ Fair ?	33.4^d^ Fair ?	71.4 Good ?	41.7^h^ Fair ?	Convergent 39.1 Fair ? Discriminant 35.3 Fair +
	Spaulding, 2012	NR	NR	NR	NR	NR	Convergent 47.8 Fair ?
TOLD-I:4	TOLD-P:4 Manual	71.4^b^ Good ?	Test-retest 72.4 Good ? Inter-rater 41.7^c^ Fair ?	40^d^ Fair ?	57.1 Fair ?	33.4^b^ Fair ?	Convergent 60.9 Good + Discriminant 35.3 Fair ?
TOLD-P:4	TOLD-I:4 Manual	71.4^b^ Good ?	Test-retest 69.0 Good ? Inter-rater 50 Fair ?	40^d^ Fair ?	57.1 Fair ?	50^b^ Fair ?	Convergent 60.9 Good + Discriminant 35.3 Fair +
WJIVOL	WJIVOL Manual	57.2^g^ Good ?	NE	40^d^ Fair ?	78.6 Excell ?	50^b^ Fair ?	Convergent 43.5 Fair + Discriminant 41.2 Fair ?

Study outcome ratings are based on Terwee et al. (2007) and Schellingerhout et al. (2011). Excellent (Excell) = 100–75.1, Good = 75–50.1, Fair = 50–25.1, and Poor = 25–0; NR, No study reported for this measurement property in this publication; NE, study not evaluated as “poor” methodological rating; +, ?, – = See Table 3;

a Uni-dimensionality of scale not checked prior to internal consistency calculation;

b Sample size for factor analysis not stated or small;

c Type of statistical analysis used unclear or inappropriate statistical analysis according to COSMIN;

d Error measurement calculated using Cronbach alpha or split-half reliability method;

e Time interval between assessment administrations not deemed appropriate;

f sample size small;

g Internal consistency calculated on split-half reliability;

h Only reported correlations between subtests (no study using factor analysis);

* *This study was also evaluated for another of the selected assessments*.

Ratings for each measurement property are shown as percentage of total points available and classified according to quartile in which percentage falls: Excellent (Excell) = 100–75.1, Good = 75–50.1, Fair = 50–25.1, and Poor = 25–0. The rating of measurement properties based on percentages of all items allows for the overall quality of a study be considered, however it also means that it was possible for studies to be rated “excellent” or “good” overall when individual items may have been rated “poor” for methodology. The footnotes in Table 8 indicate where studies were rated “excellent,” “good,” or “fair” overall, but were identified as having a “poor” rating for important items, such as: uni-dimensionality of the scale not checked prior to internal consistency calculation; sample size not stated or small; type of statistical analysis used unclear or inappropriate statistical analysis according to COSMIN; error measurement calculated using Cronbach's Alpha or split-half reliability method; time interval between assessment administrations not deemed appropriate; internal consistency calculated using split-half reliability; or correlations between subtests reported for structural validity rather than factor analysis.

Studies with COSMIN ratings of “fair” or higher were then rated on the evidence provided in the study outcome for each measurement property using the criteria as summarized in Table 3. These results are reported in Table 8 underneath the methodological rating for each assessment. As COSMIN ratings represent the overall methodological quality of assessments and outcome ratings rate studies against specific methodological criteria, it is possible for studies with good COSMIN ratings to be rated as indeterminate for study outcome due to the presence of specific but significant flaws.

The overall rating given after considering the methodological quality and outcome of all available studies (Table 8) is provided in Table 9.

**Table 9:** Level of evidence for each assessment based on Schellingerhout et al. (2011).

**Assessment**	**Internal consistency**	**Reliability**	**Error measurement**	**Content validity**	**Structural validity**	**hypothesis testing**
ACE6-11	?	?	?	?	?	++
ALL	?	?	?	+++	?	+++
CASL	?	?	?	?	?	++^*^
CELF-5	?	++	?	++	?	+++
CELF:P-2	?	?	?	++	?	+++^*^
DELV-NR	?	?	?	?	?	?^*^
ITPA-3	?	?	?	?	?	+
LCT-2	?	?	?	?	?	+
NRDLS	?	?	?	?	NA	++
OWLS-II	?	+	?	?	?	+
PLS-5	?	?	?	++	?	+++
TELD-3	?	?	?	?	?	+
TOLD-I:4	?	?	?	?	?	++
TOLD-P:4	?	?	?	?	?	++
WJIVOL	?	NA	?	?	?	+

*+++ or ——, Strong evidence positive/negative result; ++ or —-, Moderate evidence positive/negative result; + or –, Limited evidence positive/negative result; ±, Conflicting evidence across different studies; ?, Unknown due to poor methodological quality (See Table 4); NA, no information available. Blue shading, positive evidence; yellow shading, evidence unknown*.

* *Some studies outside of the manuals were rated as having conflicting evidence within the same study*.

For seven assessments, studies examining diagnostic accuracy were identified. This information came from the respective manuals and one article. Data on sensitivity, specificity, positive predictive power and negative predictive power for these seven assessments are presented in Table 10. With regards to the assessments reviewed in this study, sensitivity and specificity indicates the percentage of children with language impairment identified by the assessment as having language impairment (sensitivity) and the percentage of children with no language impairment identified as having no language impairment (specificity). Higher values indicate higher diagnostic accuracy, with literature suggesting that values between 90 and 100% (0.90–1.00) indicate “good” accuracy and values between 80 and 89% (0.80–0.89) indicate “fair” accuracy (Plante and Vance, 1994; Greenslade et al., 2009). Predictive power indicates how precise an assessment is in predicting children with language impairment (Positive Predictive Power or PPP) and children without language impairment (Negative Predictive Power or NPP) for different cut-off scores against a pre-determined prevalence base rate. Higher predictive values indicate better precision in predictive power.

**Table 10:** Diagnostic Accuracy data reported for each assessment.

**Assessment**	**Manual or article**	**Criterions**	**Sensitivity %**	**Specificity %**	**PPP %**	**NPP%**
ALL	ALL Manual	10% base rate for population sample; 50, 70, 80, and 90% base rate for referral population; Other criterion not specified	−1 SD = 98 −1.5 SD = 86 −2 SD = 54	−1SD = 89 −1.5 SD = 96 −2 SD = 98	10% base rate: −1 SD = 50 −1.5 SD = 73 −2SD = 77 80% base rate: −1 SD = 97 −1.5 SD = 99 −2 SD = 99	10% base rate: −1 SD = 100 −1.5 SD = 98 −2 SD = 95 80% base rate: −1 SD = 93 −1.5 SD = 30 −2 SD = 35
CELF-5	CELF-5 Manual	10% base rate for population sample; 50, 60, 70, and 80% base rate for referral population; Other criterion not specified	−1 SD = 100 −1.3 SD = 97 −1.5 SD = 85 −2 SD = 57	−SD = 91 −1.3 SD = 97 −1.5 SD = 99 −2 SD = 100	10% base rate: −1 SD = 55 −1.3 SD = 78 −1.5 SD = 86 −2 SD = 100 80% base rate: −1 SD = 98 −1.3 SD = 99 −1.5 SD = 100 −2 SD = 100	10% base rate: −1 SD = 100 −1.3 SD = 100 −1.5 SD = 98 −2 SD = 95 80% base rate: −1 SD = 100 −1.3 SD = 89 −1.5 SD = 62 −2 SD = 37
CELF:P-2	CELF:P-2 Manual	20% base rate for population sample; 50, 70, 80, and 90% for referral sample	NR	NR	20% base rate: −1 SD = 53 −1.5 SD = 66 −2 SD = 82 80% base rate: −1 SD = 95 −1.5 SD = 97 −2 SD = 99	20%base rate: −1 SD = 95 −1.5 SD = 91 −2 SD = 86 80%base rate: −1 SD = 57 −1.5 SD = 39 −2 SD = 28
	Eadie et al., 2014	CELF-P:2 scores at 4 years against CELF-4 scores at 5 years	−1.25 SD = 64.0 −2 SD = 42.1	−1.25 SD = 92.9 −2 SD = 98.6	NR	NR
DELV-NR	DELV-NR Manual	10% base rate for population sample; 50, 60, 70, and 80% base rate for referral population; Other criterion not specified	−1 SD = 95 −1.5 SD = 69 −2 SD = 36	−1 SD = 93 −1.5 SD = 99 −2 SD = 100	10% base rate: 1 SD = 61 −1.5 SD = 87 −2 SD = 100 80% base rate: 1 SD = 98 −1.5 SD = 100 −2 SD = 100	10% base rate: −1 SD = 99 −1.5 SD = 97 −2 SD = 93 80% base rate: 1 SD = 84 −1.5 SD = 45 −2 SD = 28
PLS-5	PLS-5 Manual	20% base rate for population sample; 50, 70, 80, and 90% for referral sample; Other criterion not specified	With standard score 85 as cut-off = 91	With standard score 85 as cut-off = 78	20% base rate: −1 SD = 51 −1.5 SD = 73 −2 SD = 78 80% base rate: −1 SD = 94 −1.5 SD = 98 −2 SD = 98	20% base rate: −1 SD = 95 −1.5 SD = 92 −2 SD = 87 80% base rate: −1 SD = 55 −1.5 SD = 41 −2 SD = 30
TOLD-I:4	TOLD-P:4 Manual	Criterion against other assessments: ^a^PLOS, ^b^PPVT-3, ^c^TOLD-P:4, ^d^WISC-IV, and ^e^Global Language score; Other criterion not specified	With Standard Score 90 as cut-off: ^e^Global Language Score = 77	With Standard Score 90 as cut-off: ^e^Global Language Score = 89	With Standard Score 90 as cut-off: ^e^Global Language Score = 71	NR
TOLD-P:4	TOLD-I:4 Manual	Criterion against other assessments: ^a^PLOS, ^f^TOLD-P:4, and ^g^Global Language Score; Other criterion not specified	With Standard Score 90 as cut-off: ^g^Global Language Score = 75	With Standard Score 90 as cut-off: ^g^Global Language Score = 87	With Standard Score 90 as cut-off: ^g^Global Language Score = 71	NR

PPP, Positive Predictive Power; NPP, Negative Predictive Power; Base rate for population sample, percentage of population expected to identify with language impairment; Base rate for referral population, percentage of children referred for assessment who identify with language impairment; NR, Not reported in this study; SD, Number of standard deviations selected as cut-off for calculation;

a PLOS, Pragmatic Language Observation Scale;

b PPVT-3, Peabody picture Vocabulary test-Third Edition;

c TOLD-P:4, Test of Oral Language Development-Primary: 4th Edition;

d WISC-IV, Weschler Intelligence Scale for Children-4th Edition (Verbal Comprehension Composite);

e Global Language Score, Metavariable combining PLOS, PPVT-3, TOLD-P:4, WISC-IV scores;

f TOLD-P:4, Test of Language Development-Intermediate: 4th Edition;

g *Global Language Score, Metavariable combining PLOS and TOLD-P:4 scores*.

It should be noted that whilst these results from diagnostic accuracy studies are reported without being rated for methodological quality, significant methodological concerns were noted and are reported in the discussion section of this study.

## Discussion

### Methodological quality of studies

In this study, a total of 121 studies across all six measurement properties were rated for methodological quality. Of these, 5 were rated as “excellent” for overall methodological quality, 55 rated as “good,” 56 rated as “fair,” and 5 rated as “poor.” However, whilst almost half (*n* = 60) of all studies rated as “good” or better overall, only one quarter (*n* = 29) of all studies had sufficient methodological quality to meet the criteria in Table 3 based on a revision of criteria proposed by Terwee et al. (2007) and Schellingerhout et al. (2011). Therefore, over half of the studies with generally good design were identified as having specific weaknesses which ultimately compromised the usefulness of findings. Methodological flaws in studies examining psychometric quality of language assessments have also been noted in other literature (LEADERS, 2014, 2015). Therefore, there is a great need for improvements in the design and reporting of studies examining psychometric quality of language assessments for children. Clinicians and researchers also need to be critical of methodology when viewing the results of studies examining reliability and validity of assessments.

Overall, across all measurement properties, reporting on missing data was insufficient, with few studies providing information on the percentage of missing items or a clear description of how missing data was handled. Bias may be introduced if missing data is not determined as being random (Bennett, 2011); therefore, this information is important when reporting on the methodology of studies examining psychometric quality.

A lack of clarity in reporting of statistical analysis was also noted, with a number of assessment manuals not clearly reporting the statistics used. For example, studies used terms such as “correlation” or “coefficient” without specifying the statistical procedure used in calculations. Where factor analysis or intra-class correlations were applied in structural validity or reliability studies, few studies reported details such as the rotational method or formula used. Lack of clear reporting creates difficulty for independent reviewers and clinicians to appraise and compare the quality of evidence presented in studies.

COSMIN ratings for *internal consistency* were rated between “excellent” and “fair” with most rated as “good.” However, only two thirds of the reviewed assessments used the statistical analysis required for evidence of internal consistency according to Terwee et al. (2007) and Schellingerhout et al. (2011); that is, Cronbach's Alpha or Kuder-Richardson Formula–20. The remaining assessments (CASL, CELF-5, OWLS-II, PLS-5, and WJIVOL) used a split-half reliability method. Of the ten studies that utilized Cronbach alpha, five studies did not have uni-dimensionality of the scale confirmed through factor analysis and the remaining five did not have an adequate sample size. For internal consistency results to have interpretable meaning, the scale needs to be identified as being uni-dimensional (Terwee et al., 2012).

With regards to *reliability* most assessments rated in the range of “good” or “fair.” Three assessments (ACE6-11, CASL, and NRDLS) reported test-retest reliability but did not examine inter-rater reliability. One assessment (WJIVOL) did not present with any reliability studies for the subtests that contribute to composite scores that target oral language. All other assessments included examinations of both test-retest and inter-rater reliability within the manuals. Two assessments (OWLS-II and TELD-3) were designed with alternate record forms and, although not included in this review, it was noted that these assessments also reported on the parallel-forms reliability. However, only two assessments (CELF-5 and OWLS-II) used the statistical analysis identified as optimal in Table 3, intra-class correlation or weighted kappa; and were thus the only two studies identified as having evidence of reliability.

COSMIN ratings for *measurement error* were rated the lowest of all measurement properties, with no studies rating better than “fair.” All studies were rated “poor” for statistical analysis as reliabilities calculated from split-half or Cronbach alpha were used to calculate standard error of measurement, which does not meet COSMIN's requirement of two administrations for evaluating measurement error (Terwee et al., 2012). Measurement error is the variability of random error that may affect assessment results. This is used to develop confidence intervals for scores and reflects the precision to which assessment scores for individuals can be reported.

Ratings for *content validity* varied considerably across different assessments. While most assessments mapped content onto modalities of comprehension and production and domains of semantics, syntax/morphology, pragmatics and phonology, different theoretical constructs were used to guide content selection. As no empirical evidence currently exists regarding the modalities or domains of language that should be assessed and the criteria for determining impairment (Tomblin et al., 1996; Tomblin and Zhang, 2006; Van Weerdenburg et al., 2006; Eadie et al., 2014), assessments that rated lower were those that did not: (1) provide a clear definition of theoretical construct, (2) provide a clear rationale for how items were selected for the purpose of the assessment, or (3) have an assessment of content from experts during the development of the assessment. The assessments identified as having evidence of content validity were the ALL, CELF-5, CELF:P-2, and PLS-5.

COSMIN ratings for *structural validity* studies rated between “good” and “poor.” Of the 15 assessments rated, nine assessments (ALL, CELF-5, CELF-P:2, ITPA-3, CASL, OWLS-II, TOLD-P:4, TOLD-I:4, WJIVOL) had an examination of structural validity using factor analysis which is the statistical method required for evidence of structural validity according to COSMIN and Schellingerhout et al. (2011). However, of these nine assessments, only two (CELF-5 and ITPA-3) were rated as “good” or “excellent” for the sample size used. Sample size for factor analysis depends on the number of items in an assessment. As comprehensive language assessments tend to have a large number of items, many studies did not have sample sizes large enough for an “excellent” factor analysis rating on COSMIN, despite the sample appearing large. No studies reported on the percentage of explained variance in structural validity studies, therefore no studies were rated as having a good level of evidence in this measurement property.

Five assessment manuals (ACE6-11, DELV-NR, LCT-2, PLS-5, and TELD-3) did not report on a structural validity study using factor analysis but reported on correlations between subtests; however, this is not sufficient evidence of structural validity according to COSMIN. One assessment (NRDLS) did not provide any evidence to support structural validity through either factor analysis or an examination of correlations between subtests. Structural validity studies are important to examine the extent to which an assessment reflects the underlying constructs being measured in both the overall score and the subtests.

The majority of studies relating to *hypothesis testing* rated as “fair” or “good” for overall methodological quality. All 15 assessments reported on a comparison between the performance of children with language impairment and typical children and all, except the LCT-2, provided information on convergent validity with related measures of language. Fourteen studies presented with some level of evidence in this measurement property, with only one study (DELV-NR) lacking in studies with sufficient methodological quality for evidence to be determined. For three assessments (CASL, CELF-P, DELV-NR) convergent validity studies outside of the manuals presented with conflicting results. However, it t should be noted that these assessments were three of the very few assessments for which independent studies were identified. As such, the possibility exists that conflicting evidence may appear for other assessments if independent studies were available.

Studies on *diagnostic accuracy* were available for half of the selected assessments. This information included studies examining positive predictive power (PPP) using estimates of the percentage of children expected to have language impairment in a sample population and studies examining sensitivity and specificity using another assessment as a criterion. Population estimates were set at 10–20% for an overall child population and 60–90% for a population of children referred to services for assessment. Many studies also included PPP calculations with a base percentage of 50%. Most assessments presented data using a range of different standard deviations as cut-off points (between 1 standard deviation and 2 standard deviations) for identification of impairment. The variation in population estimates and cut-off points may reflect the lack of consistency with criteria for diagnosis of language impairment which is noted in literature (Tomblin et al., 1996; Spaulding et al., 2006; Greenslade et al., 2009).

Diagnostic accuracy studies were not rated for methodological quality; however significant methodological flaws were noted in the reporting of information. The evaluated article (Eadie et al., 2014) reported the sample size and sample selection methods used in the study, however no manuals reported this information. When this information is lacking, it is impossible for speech pathologists to evaluate the quality of study or to determine if the sample population represents the clinical population for which the assessment is to be used (Dollaghan and Horner, 2011). Of the studies reporting on sensitivity and specificity against another criteria for identifying language impairments, only the TOLD-P:4 manual, TOLD-I:4 manual and the article (Eadie et al., 2014) provided any description of the reference measure used and time length between assessment administrations. This lack of reporting is a serious flaw as it does not allow for the impact of potential classification errors by the reference standard to be considered in evaluating the validity of findings (Dollaghan and Horner, 2011; Betz et al., 2013). When the reference standard is not specified it also creates difficulty when attempting to compare findings for different assessments or compare different studies for the same assessment. Therefore, evidence regarding the diagnostic accuracy of currently available language assessments is lacking due to an overall trend with poor methodological quality. Improvements in methodological quality and reporting of studies are needed to provide this evidence and to assist Speech Pathologists in understanding the diagnostic utility of available assessments (Dollaghan and Horner, 2011; LEADERS, 2014, 2015).

An important discovery was that all the studies examined in this review used statistical methods solely from classical test theory (CTT), as opposed to item response theory (IRT). Although some manuals made reference to the use of IRT methods in the initial development of assessment items, no studies reported any details or outcomes for these methods. Whilst COSMIN does not currently indicate a preference between these two methods, IRT methods are increasingly being utilized for the development of assessments within fields such as psychology and have numerous reported advantages over CTT-only methods (Reise et al., 2005; Edelen and Reeve, 2007). Further investigation is needed to examine reasons for the lack of IRT methods in the development of child language assessments.

### Comparison between manuals and independent studies

Comparisons between manuals and independent articles are limited to instances where studies with adequate methodology from both a manual and an article are available for a measurement property. These included three instances examining convergent validity of the CASL, CELF:P-2 and DELV-NR (Hoffman et al., 2011; Pesco and O'Neill, 2012; Kaminski et al., 2014). In all three of these examples, the articles were rated as reporting conflicting evidence whilst studies in manuals were rated as having positive evidence. Pesco and O'Neill (2012) examined the ability for DELV-NR and CELF:P-2 scores to be predicted by earlier scores on another assessment, the Language use Inventory (LUI). The study reported correlations above the 0.5 suggested by Schellingerhout et al. (2011) for one of five age groups investigated, although the authors named a significant correlation for three age groups. Kaminski et al. (2014) examined the correlation between CELF-P:2 scores and scores on an assessment called the Preschool Early Literacy Indicators (PELI). In this study, correlations between composite scores were found to be slightly above the level suggested by Schellingerhout et al. (2011) for predictive validity and slightly below for convergent validity. Another study by Hoffman et al. (2011) examined convergent validity between the CASL and the Test of Language Development-Primary: 3rd Edition (TOLD-I:3). This study identified a correlation using Pearson's *r* above the level described as acceptable by Schellingerhout et al. (2011); however, further analysis using a *t*-test for significance identified a significant difference between the composite scores from the two assessments. From this, the authors suggested that it may not be accurate to assume that different assessments can be used inter-changeably with the same results.

The correlations reported in the CELF-P:2 manual (Wiig et al., 2004) for convergent validity were higher than the correlations reported in articles, however in the manual, the CELF-P:2 was compared to different versions of itself (CELF-P and CELF-4) and with a similar test published by the same publisher (PLS-4). Therefore, the correlations would be expected to be higher than the correlations reported in the articles where the CELF-P:2 was compared to language assessments with different theoretical backgrounds. The time period between administrations of assessments also differed between studies, which may be a source of difference given the potential for change in status of children over time.

The study by Hoffman et al. (2011) also examined structural validity of the CASL using factor analysis. Although this study was not identified as having adequate methodology due to small sample size, the results are interesting to note because different findings were reported in comparison to the factor analysis reported in the CASL manual (Carrow-Woolfolk, 1999). Hoffman et al. (2011) reported evidence of a single factor model however the manual reported a 3-factor model. However, the 3-factor model was only reported in the manual for children 7 years and older, with a single factor model reported for ages six and below. The sample in the article included 6, 7, and 8 year-olds, therefore encompassing both these age-ranges. Furthermore, the two studies did not administer the same subtests from the CASL and both studies received a “poor” COSMIN rating for sample size. Factor analysis on five subtests of the CASL collectively containing 260 items would require a sample size of over 1,300 for a COSMIN rating higher than “poor,” Both these studies had sample sizes less than 250. Given the shortcomings of these studies, further studies with good methodology are required to provide evidence of structural validity.

Collectively, these findings indicate that further independent studies are required to examine the validity of different comprehensive language assessments for children. Further research is also required to determine if children are categorized similarly across different assessments with regards to diagnosis and severity of language impairment (Hoffman et al., 2011; Spaulding, 2012; Spaulding et al., 2012).

### Overall quality of language assessments

It is acknowledged that speech pathologists should consider a range of factors as well as psychometric quality when selecting an assessment for use including the clinical population for which the assessment will be used, the purpose for which the assessment will be used and theoretical construct of the assessment (Bishop and McDonald, 2009). This study examined the reliability and validity of currently available assessments and identified that all assessments present with notable shortcomings when rated against methodological quality (COSMIN) and the criteria of evaluating findings of studies (Table 3). However, considering the data that is available, some assessments have more psychometric evidence to support use as diagnostic assessments. These assessments include: ALL, CELF-5, CELF:P-2, and PLS-5. It is noted that the ALL currently only provides grade level normative data for the United States of America population. The ALL, CELF-5, and PLS-5 were all rated as having “strong” or “moderate” evidence across two or more measurement properties. The CELF:P-2 was identified as having evidence in two measurement properties from the manual, however there was some conflicting information regarding hypothesis testing in independent literature. The ALL, CELF-5, and PLS-5 were not examined in independent literature. The DELV-NR, ITPA-3, LCT-2, TELD-3, and WJIVOL had no more than limited evidence for one measurement property. However, it should be noted that where evidence is reported as lacking, it does not mean that these assessments are not valid or reliable, but rather that further research is required to determine psychometric quality.

### Implications

Standardized assessments are frequently used to make important diagnostic and management decisions for children with language impairment in both clinical and research contexts. For accurate diagnosis and provision of effective intervention, it is important that assessments chosen for use have evidence of good psychometric quality (Friberg, 2010). However, a previous study identified that speech pathologists may not be selecting child language assessments based on the psychometric quality reported in assessment manuals (Betz et al., 2013). Therefore emphasis needs to be placed on the selection of assessments that are evidence-based and appropriate to the needs of the client, the speech pathologist and the service delivery context. Speech pathologists also need to advocate for improvements to the quality of both currently used assessments and those developed in the future.

This review also identifies areas in need of further research with regards to individual assessments and development of the field of child language assessment in general. Where an assessment does not present with an “excellent” or “good” level of evidence for all measurement properties, further research is required to determine if this evidence exists. In general, further information is particularly needed to provide evidence of structural validity, measurement error and diagnostic accuracy. The use of IRT methods for statistical analysis of psychometric properties of also identified as an area in need of further exploration within the field of child language assessment.

Very limited evidence of psychometric quality currently exists outside of what is reported in manuals for child language assessments and where evidence does exist, it does not always support information reported in manuals Assessment manuals are produced by developers who have commercial interest in the assessment. Furthermore, the reporting of psychometric quality in manuals is not peer-reviewed and can only be viewed after purchasing. When assessment developers make information on psychometric properties available online or in published peer-reviewed journals, transparency is achieved and clinicians and researchers are able to review psychometric properties prior to purchasing assessments. A need for independent studies is also identified in order to provide additional information to data provided in assessment manuals. When information is able to be collated from a variety of different studies, then the evidence regarding psychometric quality of assessments will become more substantial.

This review identified a number of assessments that currently present with better evidence of psychometric quality than others, although substantially more data is required to show that any assessments have “good” evidence. Until further information becomes available, it is suggested that speech pathologists favor assessments with better evidence when assessing the language abilities of school-aged children, provided that the normative sample is appropriate for the population in which the assessment is to be used. However, given that all assessments have limitations, speech pathologists should avoid relying on the results of a single assessment. Standardized assessment results should be supplemented with information from other assessment approaches (e.g., response to intervention, curriculum-based assessment, language sampling, dynamic assessment) when making judgments regarding diagnosis and intervention needs (Hoffman et al., 2011; Eadie et al., 2014). In addition, as it is possible that differences in underlying constructs between assessments contributes to differences in diagnostic abilities of assessments (Hoffman et al., 2011), it is important for speech pathologists to consider theoretical construct when choosing standardized assessments for use or when comparing results between different assessments.

## Limitations

Due to a need to restrict size, responsiveness was not investigated in this review. It was, however, noted that no assessment manuals reported on responsiveness studies. These studies have a longitudinal design with multiple administrations of the assessment across time to measure sensitivity to change in a person's status. Evidence of responsiveness is particularly important when assessments are to be used for measuring intervention outcomes or monitoring stability over time (Eadie et al., 2014; Polit, 2015). Therefore, further research is recommended to investigate the evidence for using comprehensive language assessments for these purposes. Further investigation is also needed to compare assessments across different English speaking countries and cultural groups.

This review was confined to school-age language assessments that cover both the production and comprehension of spoken language. While this reflects current literature and clinical practice (Tomblin et al., 1996; Wiig, 2010), there may be clinical applications for assessments specific to one modality, for example when assessing language abilities of children who are non-verbal or have unintelligible speech. Assessments targeting single aspects of language such as semantics or syntax were also not included in this study, however, these may be used by Speech Pathologists (Betz et al., 2013), therefore an examination of psychometric quality of these assessments is recommended.

There is a need for future research to examine the psychometric quality of assessments for children who are bi-lingual or speaking English as a second language (Gillam et al., 2013). An examination of standardized written language assessments is also needed as there is a strong overlap between spoken and written language impairment in school-aged children (Bishop and Snowling, 2004; Snowling and Hulme, 2012). In addition, there is also a need for investigation into assessments that target activity and participation levels of the World Health Organization's International Classification of Functioning and Disability—Child and Youth (McLeod and Threats, 2008; Roulstone et al., 2012).

## Conclusion

This systematic review examines the psychometric quality of 15 currently available standardized spoken language assessments for children aged 4–12 years. Overall, limitations were noted with the methodology of studies reporting on psychometric quality, indicating a great need for improvements in the design and reporting of studies examining psychometric quality of both existing assessments and those that are developed in the future. As information on psychometric properties is primarily provided by assessment developers in manuals, further research is also recommended to provide independent evidence for psychometric quality. Whilst all assessments were identified as having notable limitations, four assessments: ALL, CELF-5, CELF:P-2, and PLS-5 were identified as currently having better evidence of reliability and validity. These four assessments are suggested for diagnostic use, provided they suit the purpose of the assessment process and are appropriate for the population being assessed. Emphasis on the psychometric quality of assessments is important for speech pathologists to make evidence-based decisions about the assessments they select when assessing the language abilities of school-aged children.

## Author contributions

DD, RS, NM, WP, and RC all contributed to the conceptual content of the manuscript. DD and YC contributed to data collection and analysis.

### Conflict of interest statement

The authors declare that the research was conducted in the absence of any commercial or financial relationships that could be construed as a potential conflict of interest.

## Footnotes

^1^Recent international consensus has replaced the term Specific Language Impairment with Developmental Language Disorder (Bishop et al., 2017).
